# KCNA2 Autoimmunity in Progressive Cognitive Impairment: Case Series and Literature Review

**DOI:** 10.3390/brainsci11010089

**Published:** 2021-01-12

**Authors:** Charles Timäus, Philipp von Gottberg, Sina Hirschel, Claudia Lange, Jens Wiltfang, Niels Hansen

**Affiliations:** 1 Department of Psychiatry and Psychotherapy, University of Goettingen, Von-Siebold-Str. 5, 37075 Goettingen, Germany; sina.hirschel@med.uni-goettingen.de (S.H.); clange@gwdg.de (C.L.); jens.wiltfang@med.uni-goettingen.de (J.W.); niels.hansen@med.uni-goettingen.de (N.H.); 2 Department of Neuroradiology, University Medical Center Goettingen, Robert-Koch-Str. 40, 37075 Goettingen, Germany; p.vongottberg@med.uni-goettingen.de; 3 German Center for Neurodegenerative Diseases (DZNE), Von-Siebold-Str. 3a, 37075 Goettingen, Germany; 4 Neurosciences and Signaling Group, Department of Medical Sciences, Institute of Biomedicine (iBiMED), University of Aveiro, 3810-193 Aveiro, Portugal

**Keywords:** autoimmunity, cognitive impairment, neural autoantibodies, KCNA2, VGKCC

## Abstract

Autoimmune dementia is a novel and expanding field which subsumes neuropsychiatric disorders with predominant cognitive impairments due to an underlying autoimmune etiology. Progressive dementias with atypical clinical presentation should trigger a thorough diagnostic approach including testing for neural surface and intracellular antibodies to avoid a delay in accurate diagnosis and initiating appropriate therapy. Here, we present two emerging cases of progressive dementia with co-existing serum autoantibodies against the KCNA2 (potassium voltage-gated channel subfamily A member 2) subunit. We found various cognitive deficits with dominant impairments in the memory domain, particularly in delayed recall. One patient presented a subacute onset of then-persisting cognitive deficits, while the other patient’s cognitive impairments progressed more chronically and fluctuated. Cognitive impairments coincided with additional neuropsychiatric symptoms. Both had a potential paraneoplastic background according to their medical history and diagnostic results. We discuss the potential role of KCNA2 autoantibodies in these patients and in general by reviewing the literature. The pathogenetic role of KCNA2 antibodies in cognitive impairment is not well delineated; clinical presentations are heterogeneous, and thus a causal link between antibodies remains questionable. Current evidence indicates an intracellular rather than extracellular epitope. We strongly suggest additional prospective studies to explore KCNA2 antibodies in specifically-defined cohorts of cognitively impaired patients via a systematic assessment of clinical, neuropsychological, neuroimaging, as well as laboratory and CSF (cerebrospinal fluid) parameters, and antibody studies to (1) determine the epitope’s location (intracellular vs. extracellular), (2) the mode of action, and (3) seek co-existing, novel pathogenetic autoantibodies in sera and CSF.

## 1. Introduction

Voltage-gated potassium channels play a pivotal role in regulating neuronal excitability and synaptic functions. 40 genes are known to encode for 12 protein subfamilies (Kv.1.-12.) of which the KCNA2 gene encode for an alpha subunit for Kv1.2 [1]. Kv1 channels consist of a (homo-/hetero-) tetrameric structure out of four alpha subunits. Combining different Kv1 subunits gives rise to a variety of different isoforms of Kv1 shaker-related ion channels exerting special electrophysiological characteristics [2]. The Kv1-subfamily is ubiquitously expressed, but at high density in CNS (central nervous system) tissue with respect to Kv1.1, Kv1.2 and Kv1.4 channels [3]. Furthermore, neural site-specific variations in the structure of Kv1.2 indicate its distinct functions within different neural compartments [4]. Neurological disorders associated with genetically altered VGKC (voltage gated potassium channel) genes, i.e., KCNA2-related disorders, are heterogeneous, ranging from epileptic encephalopathies to hereditary spastic paraplegia and episodic ataxia [5,6,7]. About two decades ago, patients with symptoms in the peripheral or central nervous system or both were increasingly found to possess antibodies labelling VGKC channels. They were thought to bind Kv1 channels and, therefore, directly interfere with their functions on an autoimmune basis. Firstly, they were described in patients with neuromyotonia [8], Morvan’s syndrome [9], limbic encephalitis [10,11] and faciobrachial dystonic seizures. Later evidence revealed that antibodies in the sera of the aforementioned patients did not react with the VGKC complex itself, but were directed to specific VGKC-associated proteins known as LGI1 (Leucin rich glioma inactivated (1) and CASPR2 (contactin-associated protein (2) [12,13]. Antibodies against LGI1 and CASPR2 were linked to well-described immunotherapy-responsive autoimmune syndromes in the peripheral and central nervous system. In contrast, a broad spectrum of VGKC-associated antibodies excluding CASPR2- and LGI1-antibodies exists that are not closely linked to specific neuropsychiatric syndromes, and their pathogenic roles are questionable [14]. In autoimmune encephalitis, as cognitive dysfunctions can dominate the onset of neuropsychiatric symptoms, they can often mimic the clinical features of typical neurodegenerative dementia [15,16]. Here we present for the first time two patients suffering predominant cognitive decline and neuropsychiatric symptoms in the presence of autoantibodies against the KCNA2 (Kv1.2) subunit of the VGKC complex. We thoroughly discuss the potential role of KCNA2 in both cases and provide recommendations for future investigations. We emphasize the importance of taking a systematic diagnostic approach in patients presenting with clinical features signaling an autoimmune-related cognitive decline that they might mimic, but must be differentiated from neurodegenerative diseases, as the therapeutic implications are immense and a delay in inducing effective immunotherapy will compromise the patient’s clinical outcome [17,18].

## 2. Case Reports

### 2.1. Case 1

**Figure 1:**
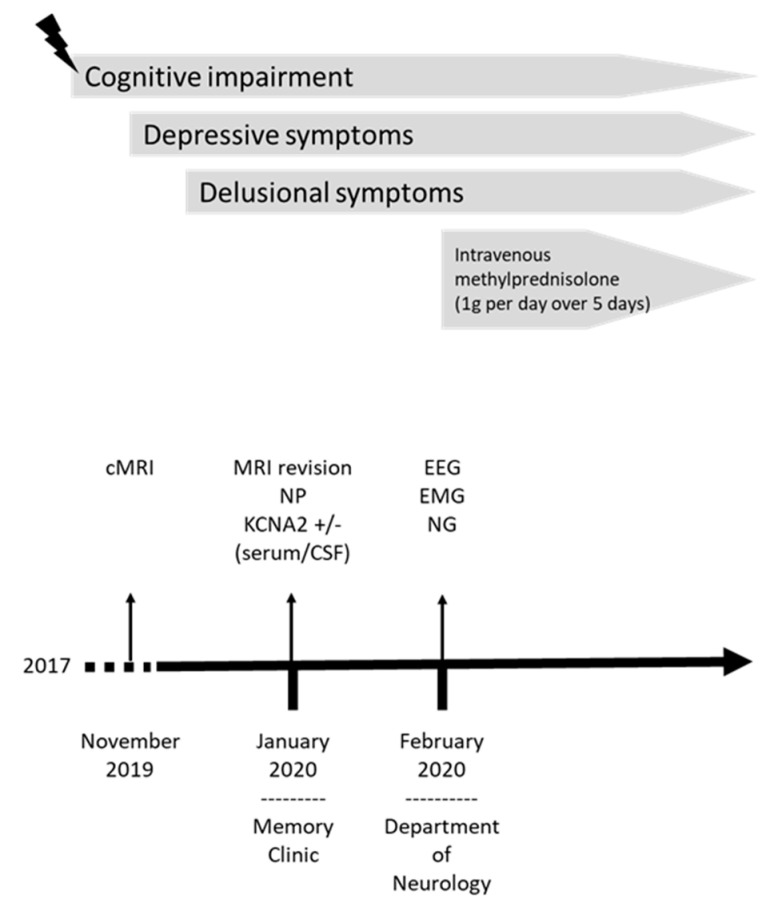
Evolution of symptoms in patient 1. The timeline comprises clinical onset, evolution of symptoms, diagnostics and treatments. The subacute onset of cognitive impairment in patient 1 is indicated by the thunderbolt. cMRI, brain magnetic resonance imaging; CSF, cerebrospinal fluid; EEG, electroencephalography; EMG, electromyography; NG, neurography; NP, neuropsychological test.

**Figure 2:**
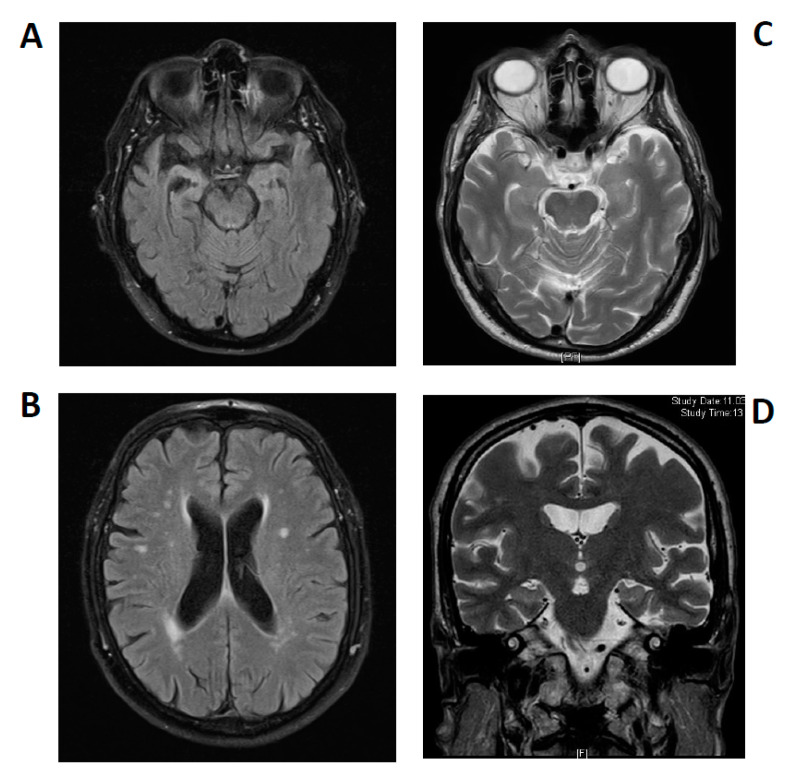
Brain magnetic resonance imaging. Patient 1: Asymmetrical atrophy of mesiotemporal structures in the right hemisphere and leukoencephalopathy (**A**,**B**): T2-weighted fluid-attenuated inversion recovery (FLAIR). Patient 2: Arachnoid cyst paramedial in posterior skull, but no further pathology (**C**,**D**) T2-weighted sequences).

A 66-year-old Caucasian male patient was first examined in January 2020 in our memory clinic (Figure 1). The patient is married and father of a daughter. He achieved an educational level of 8 years and was employed as a factory worker until retiring in 2017. His wife described a marked loss of memory functions about 3 years ago. After a subacute onset of cognitive decline, his memory problems failed to resolve, and were accompanied by depressive symptoms with insomnia over time. Symptoms of cognitive impairment were initially attributed to the affective disorder, and he was given mirtazapine at a daily dosage of 15 mg by a psychiatric consultant. The patient underwent MRI imaging of the neurocranium in November 2019, which revealed asymmetrical atrophy of the mesiotemporal region suggesting Alzheimer´s disease (Figure 2A,B). His wife also reported a progressive speech disorder, a beginning disorientation, and behavioral symptoms in the last few months. Behavioral symptoms comprised minor signs of frontal disinhibition and impulse-control dysregulation. She also reported that he had begun using swear words more often, and had developed delusional symptoms: he became convinced he was being addressed by the hosts of a certain afternoon television show and seemed to converse with them bidirectionally. The presence of hallucinations in the context of psychotic symptoms could not be confirmed. His treatment at the time of first contact in our memory clinic consisted of mirtazapine at a daily dosage of 15 mg, acetylsalicylic acid, magnesium and Vitamin D supplements. His mother grew very old (88 years), but suffered from dementia of unknown etiology. The cause of his father’s death was unknown. His medical history comprised a myocardial infarction in 2018 and intermittent muscle cramps. There were no signs of substance-related abuse, or consumption of illegal psychoactive substances.

Initial physical and neurological examination revealed no pathologies, in particular, no signs of cerebellar disorders like ataxia or oculomotor abnormalities. Routine and extended laboratory tests excluded Vitamin B1, B6, and B12 deficiency. Ceruloplasmin and cupper values in serum were normal. Thyroidal hormone levels were also normal, and screening for autoantibodies against thyroidal gland tissue was negative. Any neurological manifestation of borreliosis and lues was excluded. IgM-antibodies against borrelia burgdorferi in serum were ascribed to a persistent antibody response with no clinical relevance, as no specific borreliosis-antibodies or elevated cell count were detected in CSF. Routine CSF diagnostics continued to reveal normal values (2 cells/µL, protein 401 mg/L, oligoclonal bands negative, MRZ-VZV-HSV-AI were not elevated). Biomarkers implied no neurodegenerative disease (normal were total tau, 181pTau, ß-Amyloid 1-42, ß-Amyloid-Ratio). An anti-neural antigen immunofluorescence test exposed Anti-KCNA2 (voltage-gated potassium channel ß-subunit Kv1.2) (1:100) antibodies in serum only. No LGI1 or CASPR2 reactivity was identified (Table 1).

His MRI dataset was reviewed in the Department of Neuroradiology, confirming an asymmetrical atrophy of the mesiotemporal and right temporopolar brain regions and progressive leukoencephalopathy in white matter (Figure 2A,B). MRI data were initially compared to those obtained in 2014 that had shown no atrophy and fewer white matter lesions. The mesiotemporal atrophy was interpreted as being compatible with a limbic encephalitis at later stages, which was recently reported in courses of limbic encephalitis by Wagner et al. [19].

**Figure 3:**
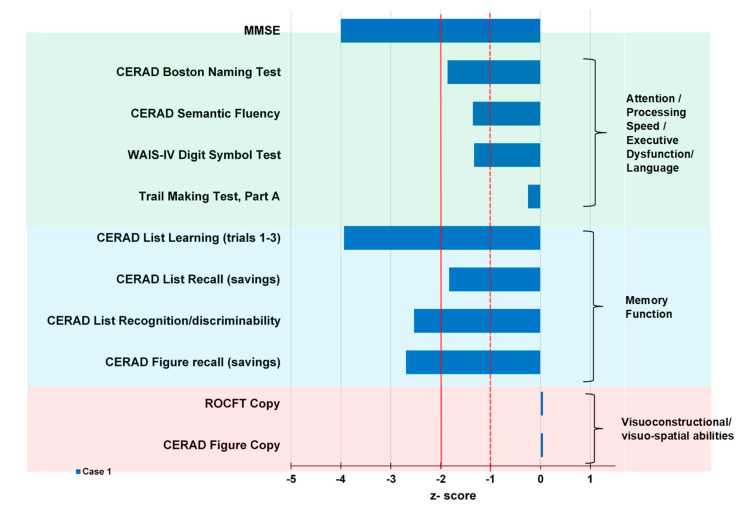
Results of neuropsychological tests of patient 1. Results are provided as z-scores enabling direct comparison with healthy subjects of the same age, gender, and education level. The dashed line indicates the cut-off for pathological scores (−1: mild deficits; −2: marked deficits). Trail Making B was not evaluated as the patient discontinued this test. CERAD, Consortium to Establish a Registry for Alzheimer’s Disease; ROCFT, Rey-Osterrieth-Complex-Figure test; WAIS-IV, Wechsler Adult Intelligence Scale IV.

Preliminary cognitive screening detected a significant result in MMSE (Mini Mental State Examination) (23/30). The clock-drawing test was normal, but comprehensive neuropsychological assessments including the CERAD (Consortium to Establish a Registry for Alzheimer’s Disease) test-plus battery (covering these cognitive domains: attention, language, visuoconstruction, memory and executive functions) and ROCFT (Rey-Osterrieth-Complex-Figure test) revealed marked deficits in figural and verbal memory parameters associated with an impaired delayed recall. Moreover, language impairments (Boston Naming Test and Semantic fluency) were moderate. His working memory was also moderately affected, whereas visuoconstructual/visuo-spatial abilities were unaffected. As our patient is not a native German-speaker, his verbal memory and language functions must be interpreted very carefully (Figure 3). Nevertheless, in light of his relevant deficits in daily-living abilities, we diagnosed a dementia.

Subsequently, the patient was admitted to the Department of Neurology in February 2020. EEG recordings revealed no abnormalities—neither epileptic potentials nor any focal slowing. Thorough electrophysiological examinations (EMG, neurography) revealed no signs of peripheral nerve hyperexcitability, nor acute or chronic denervations. Thus, Morvan´s syndrome was unlikely. Daily intravenous corticosteroids were applied over 5 days and well tolerated. His wife reported improved speech fluency and attentiveness. We screened for any malignancy, including a conventional X-ray of the thorax and ultrasound diagnostics of the abdomen. An undefined tumorous structure in the bladder and additional hyperdense lesion in the lung were identified. We recommended a further computer tomography of the thorax and abdomen and PET (positron emission tomography)-tumor-screening, but the patient refused.

### 2.2. Case 2

**Figure 4:**
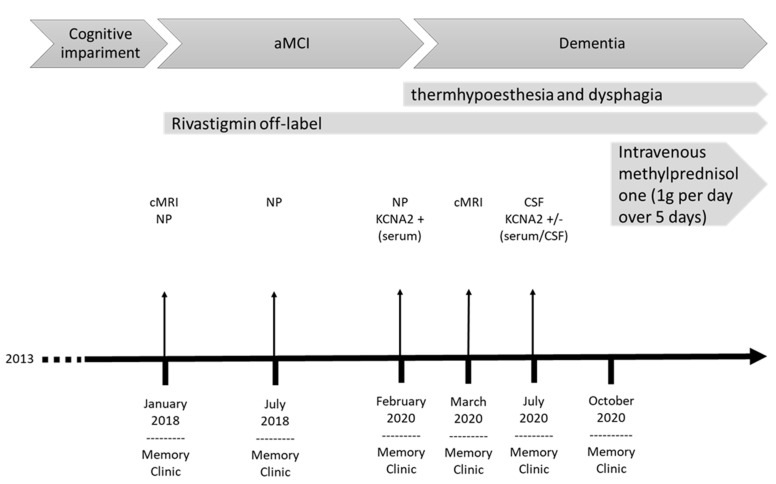
Evolution of symptoms in patient 2. The timeline comprises clinical onset, evolution of symptoms, diagnostics and treatments. Consecutive neuropsychological tests detected cognitive fluctuations in patient 2. aMCI, amnestic mild cognitive impairment; cMRI, brain magnetic resonance imaging; CSF, cerebrospinal fluid; NP, neuropsychological test.

**Figure 5:**
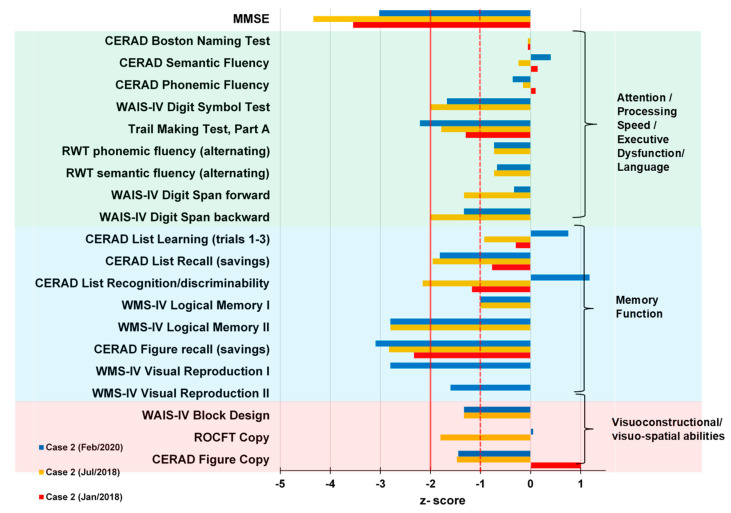
Results of neuropsychological tests of patient 2. Results are provided as z-scores enabling direct comparison with healthy subjects of the same age, gender, and education level. The dashed line indicates the cut-off for pathological scores (−1: mild deficits; −2: marked deficits). Trail Making B was not evaluated as the patient discontinued this test. CERAD, Consortium to Establish a Registry for Alzheimer’s Disease; ROCFT, Rey-Osterrieth-Complex-Figure test; RWT Regensburg word fluency test; WAIS-IV, Wechsler Adult Intelligence Scale IV; WMS-IV, Wechsler Memory-Scale IV.

A 78-year-old retired male gardener with 8 years of school education presented in our tertiary memory clinic 2018 suffering a progressive impairment of his episodic memory, concentration capacity, and word-finding difficulties since 2013 (Figure 4). He often put things in places he could not remember later. His neurological examination was unremarkable in 2018. He was married, and has a daughter. He had had colon cancer in 2010, and in 2005 bladder carcinoma, with consecutive, complete extirpation of both tumors. He had been diagnosed with borreliosis in 2000, followed by antibiotic treatment. His comorbidities were bilateral cataract and Morbus Dupuytren. No relevant neurologic or psychiatric disease was reported by his relatives. Cranial MRI revealed an arachnoid cyst paramedial in the posterior skull, but no further pathology. In 2018, an amnestic, mild cognitive impairment in multiple domains was diagnosed via CERAD testing. He had presented impaired verbal and figural memory. Consecutive tests revealed an impaired visuoconstructive capacity and working memory in 2018. No CSF analysis including serum autoantibodies testing took place in 2018. At his follow-up visit in 2020, he exhibited fluctuating difficulties with cognitive functions. We also noted psychomotor retardation. At that time, his neurological examination exhibited a distal hypoesthesia and thermohypoesthesia in the lower extremities, and dysphagia. Comprehensive neuropsychological investigation via CERAD revealed a slower processing speed (Trail Making Test, Part A), a pathological clock test, impaired working and figural memory, verbal encoding deficits related to faulty memory consolidation of complex verbal memory, a delayed recall, and impaired visuoconstructive capacity. His capacity to encode verbal material and performance in the CERAD list recognition improved (Figure 5). As his daily-life activities were significantly impaired in 2020, the dementia criteria were fulfilled. The geriatric depression scale revealed no signs of depression in 2020; his MMSE-documented cognition was better than it had been two years earlier.

Autoantibody analysis via immunofluorescence testing (due to his tumor history) as an indicator for possible underlying autoimmunity led us to detect KCNA2 autoantibodies in serum. CSF analysis 5 months later in 2020 revealed no specific autoantibodies against neural and intracellular antigens, but elevated markers of neuronal degeneration such as an elevated S100 (4.1 µg/L (pathological > 2.7 µg/L), neuron-specific enolase (35 ng/mL (pathological >30 ng/mL) and tau protein (505 pg/mL (>450 pg/mL) and a reduced ratio of Aß42/40 (0.38 (pathological ˂ 0.5). KCNA2 autoantibodies were again detected in serum 5 months after their first detection in 2020. Neuroborreliosis and lues were excluded by CSF diagnostics (Table 1). A cranial MRI 2 months after his initial presentation in 2020 revealed cerebral microangiopathy and an arachnoid cyst left paramedial in the occipital lobe, but no signs of an encephalitis (Figure 2C,D). We initiated a single course of high-dose intravenous methylprednisolone and continued rivastigmine.

## 3. Discussion

In patient 1, we described a pattern of persistent cognitive impairments ascertained through comprehensive neuropsychological assessment. His cognitive decline’s subacute onset, and the discovery of additional mood and psychotic symptoms made us suspect an autoimmune pathology, especially because of the presence of serum autoantibodies against the Kv1.2 subunit. MRI brain-images were reviewed by our neuroradiological colleagues, who confirmed the asymmetrically atrophied mesiotemporal structures as being compatible with a post-acute phase of a limbic encephalitis. A progressive reduction in hippocampal structures in the subacute stadium of limbic encephalitis was shown systematically by Wagner et al. in autoimmune-related limbic encephalitis [19]. Furthermore, in the course of limbic encephalitis, the initial swelling of hippocampal structures can be followed by their atrophy, as in the present case [20]. Progressive leukoencephalopathy in white matter was found to be compatible with demyelination or inflammation defining a possible autoimmune encephalitis, but that does not fulfill the criteria defining a definitive limbic encephalitis, which requires such alterations restricted to the medial temporal lobe [21]. In a recent position paper we emphasized that an autoimmune etiology of psychiatric syndromes including cognitive impairment is conceivable [22] despite not fulfilling the criteria for autoimmune encephalitis [23]. Current consensus articles addressing how to specify a definitive limbic encephalitis reported that at least four criteria must be met (namely, a typical subacute onset of clinical symptoms, the aforementioned bilateral MRI abnormalities, and an abnormal EEG or pathological CSF parameters and careful exclusion of alternative causes) [21,23,24] in combination with the detection of now-established antibodies against neural surface antigens or onconeural antibodies [24]. In light of the hippocampal atrophy detected here and Prüss et al. [23], we diagnosed a possible autoimmune encephalitis due to Graus criteria [21].

The atypical presentation of progressive and fluctuating cognitive impairments in conjunction with hypoesthesia, thermohypoesthesia and dysphagia, and the patient’s tumor history prompted us to take a diagnostic approach seeking potential autoimmunity in the second patient’s course, as did recently published criteria [22]. Indeed, in patient 2 we discovered KCNA2 autoantibodies corroborated by repeated measurements, and, as in patient 1, patient 2’s antibodies were restricted to serum probes. It is noteworthy that our CSF analysis revealed elevated total tau and phospho-tau 181 concentrations, which indicate axonal neurodegeneration. Furthermore, the coincidence of a reduced ß amyloid 1–42/1–40 ratio in CSF implied Alzheimer’s disease. Unlike in neurodegenerative diseases, subsequent brain MRI imaging revealed no mesiotemporal atrophy, but neither were there any specific indications for encephalitis like hyperintense lesions in mesio- and extra-temporal brain structures. Summarizing the results of CSF analyses and affection of episodic memory functions at time of onset, we discussed this case in the context of Alzheimer’s disease. According to findings by Lang et al. [14], Kv1.2 autoantibodies are likely arising secondary to diverse pathologies setting free the intracellular epitope of Kv1.2, then rendered accessible to the immune system. They stated that the primary pathological insult may not be immune. However, uncertainty remained in the presence of the KCNA2 autoantibodies, as autoimmune-mediated dementia seemed possible according to recently published criteria [22] and fluctuating cognitive deficits are known to be an indicator of a possible autoimmune dementia involving immunotherapeutic responsiveness [25]. Some authors have hypothesized that evidently elevated total tau in CSF may be associated with secondary neurodegeneration due to CNS inflammation. This mechanism was recently discussed, and CSF biomarkers of neurodegeneration have proven to correlate inversely with clinical improvement [26,27]. A potential association between autoantibodies and cognitive decline in autoimmune dementia has been thoroughly investigated, and currently recruiting clinical trials investigating the impact of immunotherapy in suspected Alzheimer’s disease associated with novel autoantibodies have been reviewed elsewhere [28,29]. Autoantibodies exhibit variable associations with paraneoplastic syndromes sometimes resembling neurocognitive syndromes such as Alzheimer’s disease, which we had to reconsider as the second patient had a positive tumor history. Actually, both men had a potential paraneoplastic background indicated by their medical history and the diagnostic findings (first patient’s). From this viewpoint, unlike well-established neuronal cell-surface antibodies (e.g., LGI1 or CASPR2), KCNA2 autoantibodies may not necessarily interfere with extracellular protein functions, but they do represent a more potential surrogate marker of a paraneoplastic neurological syndrome. Interestingly, Lang et al. recently reported paraneoplastic neuromyotonia and Lambert Eaton syndrome, both possibly related to Kv1 subunit reactivity in small-cell lung carcinoma. Unfortunately, as both patients have been lost to follow up, we have no information on further tumor diagnostics or their response to immunotherapy. Intravenous corticosteroids appeared to improve speech fluency and attention functions in the first patient, but we could not confirm this as he refused to undergo a consecutive neuropsychological assessment. We think it is probable that also non-anti-inflammatory effects, e.g., psychotropic effects, could have caused the improvement in his case. In the light of the aforementioned reports, follow-up diagnostics would undoubtedly be helpful, as there is evidence of alleviated cognitive impairments in conjunction with autoimmune dementia [25].

Overall, the pathological and clinical relevance of double negative VGCK antibodies is controversial, as they comprise a wide range of only partially known targets, heterogeneous clinical phenotypes, and reveal a variable response to standard immunotherapy [30]. KCNA2 autoantibodies were recently discovered to target the plasma membrane-associated Kv1.2 subunit in the VGKC complex of patients with suspected autoimmune neurological syndromes [31]. It is questionable if these novel autoantibodies are pathogenetic. Contrary to previous descriptions [31], recent evidence reported by Lang et al. led to the assumption that Kv1 subunit-associated autoantibodies were more likely directed to the intracellular epitope, as they did identified no detectable binding to the hippocampal cell culture system. Furthermore, they detected no specific autoimmune phenotype, and the response of most patients to immunotherapy was generally poor, with exception of one young male with Kv1.2 antibody-associated limbic encephalitis. However, they provide no detailed information on clinical factors or other diagnostic results (Table 1 in Lang et al. [14]). Discrepancies in antibody analyses of serum and CSF in our patients raised our concerns, as double-negative VGKC complex antibodies reactivity has also been found in healthy subjects [11]. Our final conclusions were driven by the clinical reasoning described above and a potential paraneoplastic association which we could not exclude (in addition to patient 2’s possible Alzheimer’s disease). 

KCNA2 autoantibodies are worthy of further study in large-scale, prospective studies including well characterized groups of patients with neurocognitive disorders. Results from previous clinical and experimental animal studies support an association between antibodies against Kv1 and impaired memory functions encoded by neuronal hippocampal circuits. In summary, these studies depicted verbal and figural memory dysfunctions either in acute [32] and in post-acute LGI1/CASPR2 or double-negative VGKC antibody-related limbic encephalitis [33,34], as does hippocampal atrophy in sequential MRIs [19,32]. Combined structural and functional MRI studies revealed disturbances in hippocampal functional connectivity predicting memory impairments rather than regional atrophy in VGKC-positive patients with post-acute limbic encephalitis [35]. Recently, the workgroup around Kirschstein et al. presented neurophysiological data showing differential effects of anti-Kv1.2 antibodies when the sera from a patient who had suffered a status epilepticus due to anti-Kv1.2-related limbic encephalitis (and who had responded well to immunotherapy) was injected in rat hippocampus. The sera of both patients with previous anti-CASPR2 encephalopathy and those with anti-Kv1.2. antibodies altered the cellular excitability of hippocampal structures in the rat, and facilitated epileptic conditions, but through different pathomechanisms [36]. Their work attributes anti-Kv1.2 with a mode of action in patients with antibody-associated temporal lobe epilepsy in limbic encephalitis, and provides evidence that anti-Kv1.2 may bear pathogenetic potential. Their conclusions make sense, as potassium voltage-gated channels are known to regulate excitability, neurotransmitter release, and synaptic functions [4,37] and genetic alterations resulting in Kv-channel dysfunctions are proven causes of neurological syndromes such as episodic ataxia and familiar epilepsy [38] as well as affection of temporal lobe structures with cognitive dysfunction and epilepsy [39]. Moreover, Kv channels are highly expressed in hippocampal neurons, which are a component in limbic circuitry known to be involved in episodic memory [40]. If this mode of action were to apply in patients with an epileptic phenotype in limbic encephalitis, we should investigate a potential contribution of Kv1.2 dysfunction in a subgroup of autoimmune related neurocognitive disorders.

In the present case series, both patients suffered various cognitive deficits and marked impairments in the memory domain, particularly in delayed recall. Patient 1 presented a subacute onset of later-persisting cognitive deficits. Together with the evidence of brain atrophy in MRI imaging and absence of neurodegenerative biomarkers, we postulated a possible autoimmune pathomechanism [23]. Patient 2’s cognitive impairments progressed more chronically and fluctuated. His CSF analyses implied Alzheimer’s disease, but we suspected an additional autoimmune contribution to his cognitive decline.

## 4. Conclusions

We present two patients exhibiting prominent memory impairments in the presence of autoantibodies against the Kv1.2 subunit. Their cognitive deficits were similar in terms of verbal and figural memory functions, but heterogenous in non-memory cognitive domains, the initial onset, and course of clinical presentation. Atypical neurological symptoms and hints of a potentially paraneoplastic origin of dementia prompted a critical review of these two patients, including the search for neural cell-surface and intracellular autoantibodies. Our diagnosis of an autoimmune disease in these patients relied on clinical reasoning including ancillary testing in our memory clinic. The role of KCNA2 autoantibodies in autoimmune-related dementia requires further investigations within large-scale, prospective study designs including well-characterized, cognitively impaired patients. Methods should include thorough clinical, neuropsychological, neuroimaging, and laboratory as well as CSF assessments to identify subgroups of dementia in whom KCNA2 autoantibodies may serve as a direct pathogenetic agent. We suggest that suitable methods should be carried out to (1) determine the antigen specificity (intracellular vs. extracellular epitopes) by using live neurons and hippocampal tissue in addition to cell-based immunofluorescence tests, and (2) search for undetected, co-existing (novel) pathogenic autoantibodies in KCNA2 positive patients’ sera and CSF. Professionals at memory clinics should be aware of the clinical features indicating a possible underlying autoimmune process in dementia, such as additional neuropsychiatric symptoms and a subacute onset or a fluctuating course of symptoms. These features should trigger a sophisticated diagnostic algorithm and search for well-established autoantibodies, as early immunosuppressive treatment can significantly improve long-term outcomes [41].

## Figures and Tables

**Table 1:** Diagnostic parameters. Analysis of specific antibodies against paraneoplastic antigens were performed by using antibody blots (Amphiphysin, CV2 (cronveinten 2), GAD65 (glutamic acid decarboxylase 65), HuD, Ma1/2, Ri, Ro, SOX1, TR, Zic4) in CSF and serum. Recombinant-cell indirect immunofluorescence was used to find antibodies against neuronal—surface antigens in CSF and serum, e.g., Aquaporin-, AMPAR1/2—(a-amino-3-hydroxy- 5-methyl-4-isoxazolepropionic acid receptor 1/2), CASPR2- (contactin-associated protein-like 2), DPPX—(dipeptidyl peptidase protein-like 6), GABAAR—(g-aminobutyric acid A receptor), LGI1—(Leucine-rich glioma inactivated 1) and NMDAR—(N-methyl-Daspartate receptor) antibodies. Analyses were performed in the CSF laboratory in the Department of Neurology, University Medical Center Goettingen, and in the Euroimmun laboratory in Luebeck, Germany. KCNA2 autoantibodies (Euroimmun) were specifically identified via a diagnostic biochip array of recombinant HEK293 cells.

Patient Number	Patient 1	Patient 2
**CSF Analysis**	**1/2020**	**7/2020**
Cell count /µL (pathological: >5 µL)	2	0
Lymphocytes in %	61	70
Monocytes in %	13	29
Plasma cells in %	-	-
Albumin mg/L	269	358
IgG mg/L	41.6	25.1
IgA mg/L	6.8	9.8
IgM mg/L	0.92	0.34
QAlb %	7.1	8.3
QIgG %	3.5	3.7
QIgA %	1.8	1.9
QIgM %	0.92	0.62
Lactat mmol/L	1.8	1.6
Oligoclonal IgG (CSF/Serum)	-/-	-/-
NSE ng/mL (pathological: >30 ng/mL)	n.a.	35
S100 µg/L (pathological: >2.7 µg/L)	n.a.	4.1
T-tau pg/mL (pathological: >450 pg/mL)	370	505
P-tau181 pg/mL (pathological: >61 pg/mL)	45	118
Aß1-42 pg/mL (pathological: ˂450 pg/mL)	1139	716
Aß1-40 pg/ml	9013	18,662
Aß ratio (pathological: ˂0.5)	1.3	0.38
**Specific antibody-indices (CSF)**		
Borreliosis-antibody-index-IgM (pathological: >1.5)	-	n.a.
Borreliosis-antibody-index-IgG (pathological: >1.5)	-	n.a.
Measles-rubella-varicella-herpes simplex-antibody-index-IgG (pathological: >1.5)	0.9	n.a.
Measles-rubella-varicella-herpes simplex-antibody-index-IgM (pathological: >1.5)	0.9	n.a.
**Anitbodies against neuronal cell-surface and paraneoplastic antigens (serum/CSF)**	**1/2020**	**2/2020 + 7/2020**
Anti-KCNA2-antibodies (pathological: >1:10)	1:100/-	1:32/-
**Serum**		
Homocysteine (pathological: >16.2 µmol/L)	10.9	16.5
Cerulopasmine (pathological: >60 mg/dL)	29.8	n.a.
Cupper (pathological: <11 µmol/L)	18.7	n.a.
Vitamin B12 (pathological: <187 ng/L)	547	365
Holotranscobalamine (pathological: <50)	194.5	n.a.
Vitamin B1 (pathological: <28 µg/L)	72.8	n.a.
Vitamin B6 (pathological: <4 µg/L)	7.4	n.a.
Folic acid (pathological: <3.1 µg/L)	12.9	10.5
CRP (pathological: >5 mg/L)	2.5	5.2
**Endocrinology Laboratory**		
Parathyroid hormone (reference range: 18.1–88.5 ng/L)	54.9	n.a.
Cortisol (reference range: 37–194)	99.4	n.a.
TSH (reference range: 0.35–4.94 mlU/L)	2.72	0.95
T3 (reference range: 1.71–3.71 ng/L)	3.34	3.27
T4 (reference range: 7.0–14.8 ng/L)	10.7	10.0
Thyroid peroxidase antibodies (pathological: >6 IU/mL)	<3	n.a.
Thyroglobulin antibodies (pathological: >14 IU/mL)	<4	n.a.
Thyroid-stimulating hormone receptor (pathological: >1.75 IU/L)	<0.80	n.a.
**Serological Diagnostics**		
Borreliosis-IgM-EIA (pathological: >9)	4.8	1.5
Borreliosis-IgG-EIA (pathological: >9)	37	15.10
Borreliosis-IgM-Immunoblot	-	-
Borreliosis-IgG-Immunoblot	+	-
Lues TPPA (pathological: >1:80)	-	-
Lues TPPA (pathological: >1:1)	-	-

- = not present; n.a.= not available; Aß ratio, ß amyloid 1-42/1-40 ratio; Aß1-40, ß amyloid 1-40; Aß1-42, ß amyloid 1-42; CRP, c-reactive protein; CSF, cerebrospinal fluid; EIA, enzyme immunoassay; IgA, immunoglobulin A; IgG, immunoglobulin G; IgM, immunoglobulin M; NSE, neuron-specific enolase; P-tau181, phosphorylated tau protein 181; QAlb, quotient albumin; QIgA, quotient immunoglobulin A; QIgG, quotient immunoglobulin G; QIgM, quotient immunoglobulin M; TPPA, treponema pallidum particle agglutination assay; TSH, thyroid-stimulating hormone; T-tau, total tau protein.

## Data Availability

The data presented in this study are available on reasonable request from the corresponding author.
